# Receipt of infant HIV DNA PCR test results is associated with a reduction in retention of HIV-exposed infants in integrated HIV care and healthcare services: a quantitative sub-study nested within a cluster randomised trial in rural Malawi

**DOI:** 10.1186/s12889-020-09973-y

**Published:** 2020-12-07

**Authors:** Elasma Milanzi, Victor Mwapasa, Jessica Joseph, Aurelie Jousset, Timothy Tchereni, Andrews Gunda, Jennipher Phiri, Jeanette C. Reece

**Affiliations:** 1 Centre for Epidemiology and Biostatistics, Melbourne School of Population and Global Health, The University of Melbourne, Carlton, Victoria Australia; 2 Victorian Centre for Biostatistics, Melbourne, Victoria Australia; 3 College of Medicine, University of Malawi, Blantyre, Malawi; 4 Clinton Health Access Initiative (CHAI), MA Boston, USA; 5 Clinton Health Access Initiative (CHAI), Lilongwe, Malawi; 6 The University of Melbourne Centre for Cancer Research, The University of Melbourne, Parkville, Victoria Australia

**Keywords:** PRIME, HIV, Retention, HIV PCR test, Mother-pair infants, Mother-to-child-transmission, Malawi

## Abstract

**Background:**

Retention of HIV-infected mothers in integrated HIV and healthcare facilities is effective at reducing mother-to-child-transmission (MTCT) of HIV. In the context of Option B+, we examined maternal and HIV-exposed infant retention across three study arms to 18 months postpartum: mother-and-infant clinics (MIP), MIP with short-messaging service (MIP + SMS) and standard of care (SOC). In particular, we focused on the impact of mothers receiving an infant’s HIV PCR test result on maternal and infant study retention.

**Methods:**

A quantitative sub-study nested within a cluster randomised trial undertaken between May 2013 and August 2016 across 30 healthcare facilities in rural Malawi enrolling HIV-infected pregnant mothers and HIV-exposed infants on delivery, was performed. Survival probabilities of maternal and HIV-exposed infant study retention was estimated using Kaplan-Meier curves. Associations between mother’s receiving an infant’s HIV test result and in particular, an infant’s HIV-positive result on maternal and infant study retention were modelled using time-varying multivariate Cox regression.

**Results:**

Four hundred sixty-one, 493, and 396 HIV-infected women and 386, 399, and 300 HIV-exposed infants were enrolled across study arms; MIP, MIP + SMS and SOC, respectively. A total of 47.5% of mothers received their infant’s HIV test results < 5 months postpartum. Receiving an infant’s HIV result by mothers was associated with a 70% increase in infant non-retention in the study compared with not receiving an infant’s result (HR = 1.70; *P*-value< 0.001). Receiving a HIV-positive result was associated with 3.12 times reduced infant retention compared with a HIV-negative result (*P*-value< 0.001). Of the infants with a HIV-negative test result, 87% were breastfed at their final study follow-up.

**Conclusions:**

Receiving an infant’s HIV test result was a driving factor for reduced infant study retention, especially an infant’s HIV-positive test result. As most HIV-negative infants were still breastfed at their last follow-up, this indicates a large proportion of HIV-exposed infants were potentially at future risk of MTCT of HIV via breastfeeding but were unlikely to undergo follow-up HIV testing after breastfeeding cessation. Future studies to identify and address underlying factors associated with infant HIV testing and reduced infant retention could potentially improve infant retention in HIV/healthcare facilities.

**Trial registration:**

Pan African Clinical Trial Registry: PACTR201312000678196.

**Supplementary Information:**

The online version contains supplementary material available at 10.1186/s12889-020-09973-y.

## Background

Since the introduction of Option B+ involving prompt initiation of ART in HIV+ pregnant and breastfeeding women regardless of clinical stage in 2011, [1] mother-to-child transmission (MTCT) of HIV has dramatically decreased [2, 3]. In the first 3 years of Option B+ implementation, new infant HIV infections in Malawi were reduced by 66% [4]. More recently, new infant infections decreased from ~ 16,000 in 2010 to ~ 4900 in 2017, with 92% of HIV+ pregnant women reported to have access to ART [5]. Some countries (Armenia, Belarus, Cuba, the Caribbean, Thailand and Malaysia) have achieved “zero MTCT” due to adherence to well-coordinated integrated services (antenatal, immunisation, paediatric care, community healthcare and HIV healthcare and surveillance programs [3, 6, 7]. In contrast, in many sub-Saharan African countries such as Malawi, poor retention to integrated services in resource-limited countries is thought to play a significant role in the failure to achieve “zero MTCT” [1, 8].

The cluster randomised controlled trial, Promoting Retention among Infants and Mothers Effectively (PRIME), conducted in rural Malawi attempted to identify and implement innovations to decrease maternal and infant drop-out in integrated neonatal, maternal and child health and HIV services [9]. This trial compared mother and infant retention between the “standard of care” (SOC) involving separate HIV-infected mother and HIV-exposed infant visits to HIV and healthcare services which is routinely implemented across sub-Saharan Africa and two interventions aimed to reduce maternal and infant drop-out in integrated services: 1) Mother-Infant Pair (MIP) clinics consisting of synchronised HIV-infected mother and HIV-exposed infant visits; and 2) MIP plus short-messaging service (SMS) to community-based volunteers following missed scheduled visits (MIP + SMS) [9].

Previous multivariate analysis of PRIME data found no difference in retention across study arms, with 19.0, 24.9 and 22.2% maternal retention and 8.0, 19.5 and 9.8% infant retention for MIP, MIP + SMS and SOC at 12 months postpartum, respectively [10]. However, Malawi modified national guidelines recommended mothers continue breastfeeding to 24 months postpartum in 2014, [11] thereby potentially exposing HIV-exposed infants to HIV infection beyond 12 months postpartum. Examining mother and infant study retention in integrated services for the duration of the study is important, considering the increased risk of MTCT from continual breastfeeding.

Moreover, examining factors associated with reduced maternal and infant retention in integrated services enables areas to be identified and future strategies targeted to improve retention. In particular, infant HIV testing throughout sub-Saharan Africa has been associated with reduced HIV-exposed infant retention in integrated services, with a systematic review reporting that Malawi had the highest rates of HIV-exposed infants drop-out in sub-Saharan Africa [12–14]. However, previous studies have not examined the effect of infant HIV PCR testing on maternal and HIV-exposed infant retention in integrated services using robust statistical modelling. Likewise, examining the impact of receiving the outcome of an infant’s HIV test result on retention in integrated care has not previously been examined. However, these analyses are clinically relevant as retaining breastfed HIV negative HIV-exposed infants in integrated HIV services ensures HIV-exposed infants are re-tested for HIV in the future due to the continued risk of MTCT of HIV via breastfeeding. It is also important to retain HIV-positive infants in integrated care to ensure infants have access to continual paediatric ART in order to prevent highly-prevalent HIV-related mortality in untreated infants less than 24 months of age [15–17].

This present sub-study nested within the PRIME cluster randomized control trial aimed to examine retention of mothers and infants in the study until the completion of the trial at 18 months postpartum. We also determined the impact of mothers receiving an infant’s HIV PCR test result, in particular, an infant’s HIV-positive test result, on maternal and infant study retention.

## Methods

### Study design and study site

The PRIME cluster randomised trial (CRT) was conducted and reported on the basis of Consolidated Standards of Reporting Trials (CONSORT) 2010 statement for cluster randomised trials [18, 19]. The trial was performed between May 2013 and August 2016 across 30 healthcare facilities in rural and semirural Malawi (protocol previously outlined) [9, 20]. All HIV-infected pregnant mothers in the districts of Salima and Mangochi across the Central and South regions of Malawi, respectively, and their infants on delivery were enrolled in PRIME, with clustering occurring from different health facilities stratified by region. Participants were randomly allocated across three study arms: mother-and-infant clinics (MIP), MIP with short-text reminders (MIP + SMS) and standard of care (SOC). Study exclusions included temporary residents or non-consenting participants.

We performed a quantitative sub-study nested within the PRIME study aimed at determining the impact of mothers receiving an infant’s HIV PCR test result on maternal and HIV-exposed infant retention in integrated healthcare facilities. We also examined maternal and HIV-exposed infant retention across study arms up until the end of the trial at 18 months postpartum: MIP, MIP + SMS and SOC.

The study design, study interventions and primary findings of the PRIME CRT have previously been reported [9, 10]. The three study arms of health care delivery are summarized in Supplementary Table 1. Briefly, the SOC involved routine Maternal, Neonatal and Child Health services comprising general antenatal and postnatal services; health education, family planning, nutritional and infant-feeding counselling integrated HIV-related services; counselling, ART initiation and follow-up and psychosocial support, with some family planning services. Infant HIV-related healthcare services included age-appropriate HIV testing (DNA PCR testing and rapid serology testing for HIV antibodies), paediatric ART treatment initiation and follow-up for infants diagnosed as HIV-positive and cotrimoxazole and isoniazid refills and immunisation and growth monitoring.

Of the treatment arms, SOC offered integrated healthcare services at different locations, often at different times, including on different days of the week and represents the standard method of care throughout Malawi, and other sub-Sahara African countries [20]. Conversely, MIP clinics offered integrated services to HIV-infected mothers and their infants in the same location on prescheduled days, with the MIP + SMS arm comprising MIP plus electronic text messages to community-based volunteers following missed scheduled visits.

We defined non-retention in the study as no longer attending *any* scheduled visits. That is, non-retention was the converse of retention in the study, with retention defined as documented evidence of mothers and infants attending all scheduled visits. While we acknowledge there are other possible definitions of retention, this definition corresponded to a clinically relevant definition for which there was extensive and available data.

### Data collection

Data collection of the PRIME CRT has previously been reported [9, 10]. Infants had HIV PCR testing < 6 months postpartum which was performed by the Ministry of Health (MoH) and transposed into the PRIME dataset. Recorded infant HIV PCR testing data in the PRIME dataset also underwent multiple stringent quality control checks against data from the MoH testing facilities. Data collection of HIV PCR test results was clearly delineated from rapid testing results. Moreover, infants only had HIV rapid testing > 12 months postpartum and all positive rapid testing results were confirmed by follow-up HIV PCR tests. Consequently, it is highly unlikely that HIV rapid testing data had any influence on the recording of HIV PCR testing data.

Only infant HIV PCR testing data were used in statistical analyses. Infant HIV PCR testing data included the date an infant’s dried blood spot was taken, details of the infant’s HIV PCR test result (date and outcome) and the date an infant’s HIV test result was given to the mother. Of note, mothers received infant HIV test results regardless of whether or not the infant was present at the appointment or had dropped out of the study. However, this meant that these infants could not receive recommended interventions such as cotrimoxazole prophylaxis and nutritional assessments.

Breastfeeding status for all participants was also recorded at each postnatal infant visit but the date of the cessation of breastfeeding was not recorded.

### Statistical methods

#### Outcome

Previously-reported PRIME primary outcomes examined the proportions of (i) antenatally-enrolled women retained in care to 12 months postpartum receiving all antiretroviral drug refills ≤14 days of scheduled refill dates and (ii) HIV-exposed infant attending 12-month scheduled visits plus ≥5/6 preceding scheduled visits and dried blood spot samples at 2 months for HIV PCR testing [9, 10].

We re-analysed PRIME data that included antenatally-enrolled women (*n* = 1072) with associated infant data (*n* = 1067). Notably, there were 18 fewer infants due to stillbirths and infant death in the first few days.

In the secondary analysis, the outcome of interest was time to study “non-retention”. Study non-retention was defined as having all of the following; (i) last recorded attendance to a scheduled clinic visit was before 17 months postpartum, (ii) next scheduled visit after the last recorded attendance was before 18 months postpartum (iii) there was no record of transfer to other health facilities or death. Subjects were censored (were not retained in the study) if one of the following was true; (i) their last recorded clinic attendance was at least 17 months postpartum (ii) scheduled visit after the last recorded attendance was after 18 months postpartum (iii) participants were transferred to other facilities that were not part of the PRIME study. This definition does not take into account the number of visits, for example a participant who attended the first five visits would be classified as “drop out” and one who only attended the last two visits would be censored. Thus, our approach considers participants not dropping out of the study as a critical aspect in HIV patient care and that patients who completely drop-out are at higher risk than those who show up to some scheduled appointments. Of note, analysis of maternal and infant drop-out was only performed using data collected as part of the PRIME study as data containing information on participants that were transferred to or accessed health care at other facilities that were not part of the PRIME study was not available for analysis.

#### Exposures

The study arms (MIP, MIP + SMS and SOC) were the main exposure in the original study. However, the main interest of our analysis was to assess the effect of (i) mothers receiving an infant’s HIV PCR test result, regardless of the test result, and (ii) mothers receiving an infant’s *HIV+* test result on survival of mothers and infants in the study (where survival referred to not dropping out of the study). Notably, while data were available for both HIV rapid testing and HIV PCR testing, we only examined the effect of HIV PCR test results on study retention as PCR testing is the recommended test for early infant HIV diagnosis as rapid testing is unable to reliably diagnose infant HIV infection due to the presence of maternal antibodies up to 18 months postpartum [21, 22]. HIV PCR testing is also the most widely-implemented test in resource-limited countries, with Malawian guidelines recommending testing at 2 months postpartum. Other variables examined included the effect of maternal education, parity, marital status, ART status prior to delivery, distance to clinic, infant’s gender, breastfeeding, gestational age at birth and delivery location on maternal and infant retention in the study.

### Statistical analysis

Summary statistics were visualised by Kaplan-Meier (KM) plots [23]. The effect of covariates examined (maternal marital status, level of education, distance to facility, mode of transport, parity and pre-study ART, and infant gender as listed in Table 1) on the hazard of maternal and infant not being retained in integrated care was estimated using a multivariate Cox PH regression model. Since the study arm was the original main exposure, we included the study arm in all analyses. Clustering from health facilities was accounted for using robust standard errors.

**Table 1:** Baseline characteristics

**a) Maternal characteristics.**			
***n*** **= 1072**	**MIP**^**a**^ **(*n*** **= 386)**	**MIP + SMS**^**b**^ **(*n*** **= 383)**	**SOC**^**c**^ **(*n*** **= 303)**
Parity median (IQR)	3 (2–4)	3 (2–5)	3 (2–4)
**Marital status n (%)**			
Single	17 (4.4)	16 (4.2)	3 (1.0)
Married	351 (90.9)	359 (93.3)	290 (94.8)
Widowed	4 (1.0)	0	2 (0.7)
Divorced	13 (3.4)	8 (2.1)	11 (3.6)
**Education level n (%)**			
None	125 (32.3)	141 (36.8)	128 (41.8)
Some Primary	194 (50.3)	202 (52.7)	133 (43.5)
Completed primary	14 (3.6)	10 (2.6)	15 (4.9)
Some secondary	41 (10.6)	24 (6.3)	24 (7.8)
Completed secondary	8 (2.1)	5 (1.3)	5 (1.6)
More than secondary	3 (0.8)	1 (0.3)	1 (0.3)
**Distance to facility n (%)**			
Less than 2 km	96 (24.9)	99 (25.9)	68 (22.2)
Between 2 and 5 km	159 (41.2)	160 (41.8)	118 (38.6)
More than 5 km	130 (33.7)	124 (32.4)	120 (39.2)
**Transport n (%)**			
Walking	233 (60.4)	247 (64.5)	239 (78.1)
Bicycle	106 (27.5)	67 (17.5)	48 (15.7)
Bus	14 (3.6)	41 (10.7)	6 (2.0)
Taxi	14 (3.6)	6 (1.6)	1 (0.3)
Motorbike	15 (3.9)	21 (5.5)	11 (3.6)
Other	3 (0.8)	1 (0.3)	1 (0.3)
**On ART prior to study n (%)**			
No, newly initiated	117 (30.3)	138 (36.0)	143 (46.7)
No, not known	46 (11.9)	32 (8.6)	18 (5.9)
Yes	222 (57.5)	213 (55.6)	145 (47.4)
**b) Infant characteristics.**			
***n*** **= 1067**	**MIP** **(*n*** **= 379)**	**MIP + SMS** **(*n*** **= 390)**	**SOC** **(*n*** **= 298)**
Gestational age, weeks: mean (sd)	37.0 (1.9)	36.9 (1.6)	37.1 (1.7)
**Delivery location n (%)**			
Health facility	328 (84.1)	328 (84.1)	245 (82.2)
Home	21 (5.5)	25 (6.4)	21 (7.1)
**Sex n (%)**			
Male	177 (46.7)	200 (51.3)	156 (52.4)
Female	202 (53.3)	190 (48.7)	142 (47.7)

^a^Mother-and-infant clinics

^b^ Mother-and-infant clinics with short messaging service

^c^Standard of care

Note: Where percentages don’t add up to 100%, the remaining percentages consisted of missing values

The effect of mothers receiving an infant’s HIV PCR test result and the hazard of maternal and infant non-retention was estimated using a time-varying multivariate Cox PH model, as we assessed receipt of an infant’s HIV test result to be a time-varying covariate. That is, mothers commenced in the study without having received the infant’s HIV test result, with this status only changing when the mother received the infant’s HIV test result, thereby violating the proportional hazard assumption. Note, that an infant’s test result was not received by mothers either because they were not available (that is, they were lost in the system), or the health facility did not have the opportunity to relay the results to the carer. Similarly, the second exposure (an infant’s HIV-positive test result) was assumed negative until the mother received a HIV-positive result. Both of these time-varying covariates were accommodated using a counting process approach to capture the time the status of either exposure changed: That is, 1) mothers received the infant’s HIV result (from no to yes) or 2) the infant’s HIV status (from negative to positive) [24]. Statistical analyses were performed in Stata 15 [25].

## Results

### Baseline characteristics

Maternal and infant baseline characteristics are presented in Table 1a and b, respectively. Four hundred sixty-one, 493, and 396 HIV-infected women and 386, 399, and 300 HIV-exposed infants were enrolled across study arms; MIP, MIP + SMS and SOC, respectively. Mothers had similar characteristics in terms of marital status and proximity to health facilities across study arms. However, women in the SOC arm were more likely to access health facility by walking and to be uneducated compared with those in the MIP + SMS and MIP arms. HIV-exposed infants had similar baseline characteristics except for the MIP arm which had a higher proportion of female infants than both MIP + SMS and SOC arms.

### Coverage of HIV DNA PCR testing among HIV-exposed infant

We found 757 infants (71.0%) had their first HIV test < 18 months postpartum, with 76.7% testing in MIP + SMS arm, 72.0% testing in MIP and 62.1% testing in SOC (Supplementary Table 3). Data documenting taking infant’s dried blood spot were not available for 310 HIV-exposed infants (29.0% of the total HIV-exposed infant cohort). Further, of the 757 infants that had dried blood spots taken, HIV test results were only available for 682 HIV-exposed infants (63.9% of the total HIV-exposed infant cohort).

**Fig. 1:**
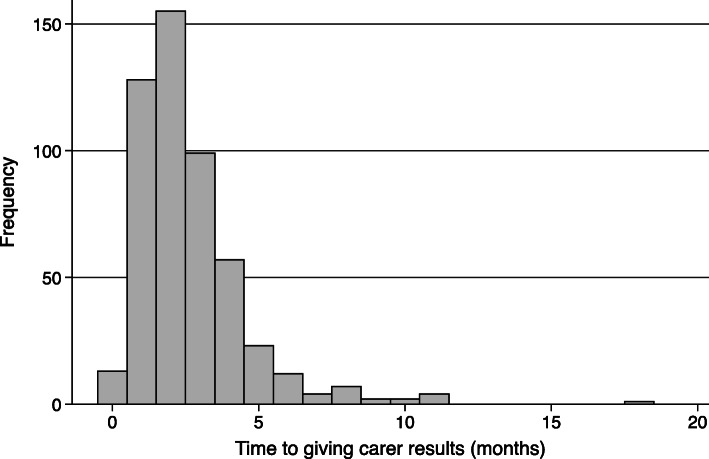
Time to mothers receiving HIV PCR blood test results since collection of infant dried blood spot (*n* = 507 mothers)

Only 507 mothers received their infant’s HIV test result (74.3% of the total infant HIV test results available and 47.5% of the total HIV-exposed infant cohort). Receiving of infant’s HIV test results was evenly distributed across different healthcare centres. The median turn-around-time between taking an infant’s dried blood spot and mothers receiving the infant’s HIV result was 2 (IQR 1–3) months and generally occurred by 5 months for most infants (Fig. 1).

### Non-retention rates in mothers and HIV-exposed infants

**Fig. 2:**
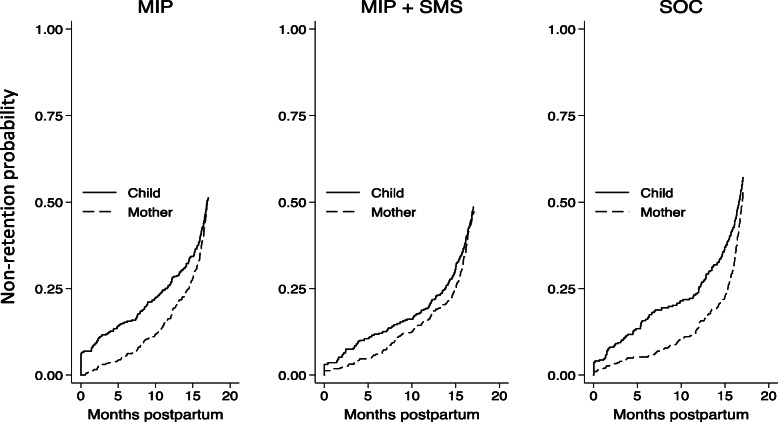
Kaplan Meier curves comparing study non-retention rates of mothers and infants in each study arm

Kaplan-Meier (KM) failure curves were used to compare maternal and infant retention across study arms (Fig. 2). Non-retention rates increased progressively throughout the postpartum period. HIV-exposed infant non-retention also occurred earlier and at a faster rate than HIV-infected maternal non-retention across all study arms, as indicated by the steeper KM curves (solid line). At 6 and 12 months postpartum, the cumulative probability of non-retention was 14 and 24% for infants and 5 and 15% for mothers, respectively. At the end of the study (18 months postpartum), infant and mother retention was 49 and 50%, respectively.

**Fig. 3:**
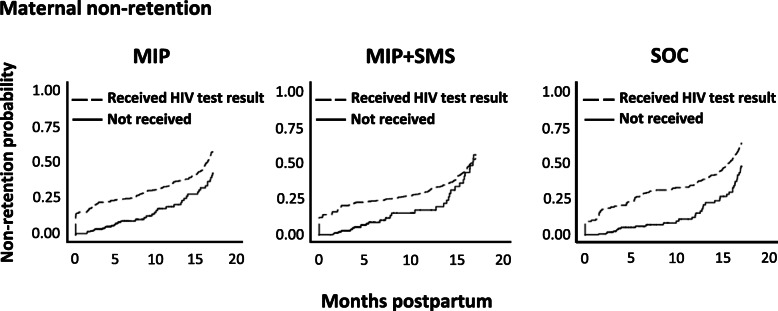
Kaplan Meier failure curves comparing study non-retention rates of mothers in each study arm by whether a mother received the infant’s HIV PCR test result. Dashed lines represent mothers that received their infant’s HIV PCR test result. Solid lines represent mothers that did not receive their infant’s HIV PCR test result

**Fig. 4:**
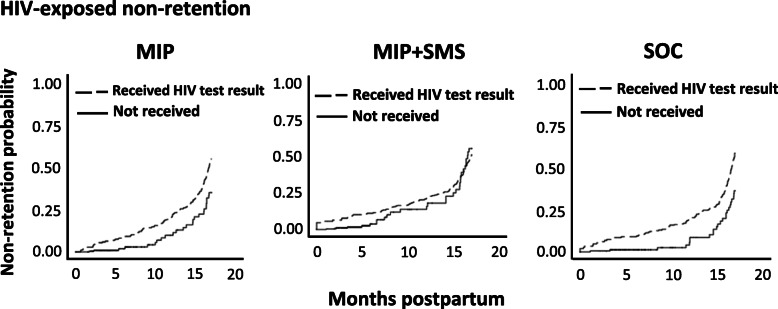
Kaplan Meier failure curves comparing study non-retention rates of infants in each study arm by whether a mother received the infant’s HIV PCR test result. Dashed lines represent mothers that received their infant’s HIV PCR test result. Solid lines represent mothers that did not receive their infant’s HIV PCR test result

To determine the impact of mothers receiving their infant’s HIV test result on maternal and HIV-exposed infant retention, we compared KM curves for mothers receiving an infant’s HIV test result with mothers not receiving an infant’s HIV test result across study arms. These curves suggested maternal (Fig. 3) and infant (Fig. 4) retention was lower across all arms when mothers received an infant’s HIV test result (dashed) compared with mothers not receiving the test result (solid). Overall, infant retention was lower than mother retention, with the greatest difference in study non-retention between infants and mothers occurring in SOC. As mentioned, only the impact of receiving an infant’s HIV PCR test results on study retention was measured, not rapid testing results due to the enhanced reliability of PCR testing for detecting HIV infection in HIV-exposed infants compared with rapid testing [21, 22]. Moreover, rapid testing in infants was only performed after 12 months postpartum.

### Covariates associated with infant study non-retention

Results from the multivariate Cox model estimating the hazard of infant non-retention (Table 2), provided some evidence to suggest MIP + SMS and MIP improved infant study retention compared with SOC by reducing non-retention by 29% (*P*-value = 0.075) and 14% (P-value = 0.445), respectively. However, differences between the two intervention arms and SOC were not statistically significant. Further, infants whose mothers commenced ART prior to enrolling in the study had 20% lower non-retention compared with those newly initiated on ART (HR = 0.80; 95%CI 0.69–0.92; *P*-value = 0.002).

**Table 2:** Time-varying multivariate Cox regression model assessing the effect of intervention and mothers receiving infant’s HIV PCR test results on the hazard of maternal and infant non-retention in the study

Parameter	Test	Infant		Mother	
		Hazard ratio (95%CI)	***P***-value	Hazard ratio (95%CI)	***P***-value
Receiving HIV test result (*n* = 682)^d^	HIV result given to mother (at any time)	**1.70 (1.30,2.30)**	**< 0.001**	1.30 (0.93,1.80)	0.118
Study arm	MIP^a^ vs SOC^b^	0.87 (0.60,1.30)	0.445	0.99 (0.64,1.60)	0.932
	MIP + SMS^c^ vs SOC	0.71 (0.49,1.00)	0.075	0.94 (0.61,1.40)	0.769
Sex (child)	Female	1.10 (0.84,1.30)	0.620	1.10 (0.83,1.31)	0.655
Parity	No. children	0.98 (0.91,1.00)	0.320	0.98 (0.91,1.11)	0.574
Marital status	Single	Ref.	Ref.	*Ref.*	*Ref.*
	Married	1.20 (0.63,2.40)	0.558	1.60 (0.69,3.70)	0.274
	Widowed	0.59 (0.03,11.0)	0.724	4.20 (0.48,38.0)	0.196
	Divorced	1.20 (0.43,3.30)	0.744	1.1 (0.31,3.81)	0.911
Education level^e^	None	*Ref.*	*Ref.*	*Ref.*	*Ref.*
	Some primary	0.94 (0.74,1.20)	0.582	0.82 (0.66,1.00)	0.069
	Completed primary	0.75 (0.40,1.40)	0.385	**0.38 (0.16,0.92)**	**0.033**
	Some secondary	0.82 (0.46,1.50	0.506	0.78 (0.49,1.20)	0.297
	Completed secondary	1.97 (0.41,2.30	0.953	0.72 (0.29,1.80)	0.472
Distance to the clinic	Less than 2 km 2-5 km	*Ref.* 0.95 (0.69,1.30)	*Ref.* 0.759	*Ref.* 0.95 (0.73,1.20)	*Ref.* 0.692
	> 5 km	1.00 (0.74,1.40)	0.886	0.89 (0.67,1.20)	0.395
Mode of transport	Walking	*Ref.*	*Ref.*	*Ref.*	*Ref.*
	Bus	0.96 (0.62,1.50)	0.843	1.35 (0.88, 1.90)	0.195
	Taxi	0.50 (0.06,4.10)	0.521	0.85 (0.24,3.10)	0.802
	Motorbike	1.20 (0.81,1.90)	0.325	0.99 (0.54,1.80)	0.988
	Other	1.70 (0.77,3.70)	0.195	2.20 (0.82,5.90)	0.115
Mother on ART prior to study	No, newly initiated	*Ref.*	*Ref.*	*Ref.*	*Ref.*
	No, not known	0.64 (0.37,1.10)	0.102	0.64 (0.33,1.20)	0.191
	Yes	**0.65 (0.49,0.85)**	**0.002**	**0.80 (0.64,0.99)**	**0.043**

^a^Mother-and-infant clinics

^b^Standard of care

^c^Mother-and-infant clinics with short messaging service

^d^The mother receiving the HIV PCR test result was treated as a time-varying covariate

^e^The number of mothers with greater than secondary education was too small to estimate the hazard ratio

### Covariates associated with maternal study retention

Multivariate Cox modelling estimating the hazard of maternal non-retention also found mothers that commenced ART prior to study enrolment were at reduced hazard of non-retention compared with mothers that had newly initiated ART (HR = 0.80; 95%CI 0.64–0.99; *P*-value = 0.043). Mothers that completed primary education had the greatest study retention (Table 2; HR = 0.38; 95%CI 0.16–0.92; *P*-value = 0.033).

### HIV testing and overall retention in the study

A time-varying multivariate Cox model was used to estimate the effect of mothers receiving an infant’s HIV test result on the hazard of non-retention in the study (Table 2). Mothers receiving an infant’s HIV result was not associated with maternal retention (HR = 1.30, *P*-value = 0.118). On the other hand, infant non-retention was 70% higher for mothers that received their infant’s HIV test result at any given time, compared with mothers that did not receive their infant’s HIV test result (HR = 1.70; 95%CI 1.30–2.30; *P*-value< 0.001).

### HIV PCR test outcome and overall retention in the study

To assess the impact of mothers receiving an infant’s HIV+ test result on maternal and infant retention, we performed a subgroup analysis on a restricted cohort of 507 mothers that received their infant’s HIV test result. (Table 3). At any given time, the hazard of non-retention was 3-fold higher for infants that tested HIV-positive compared with infants that tested HIV-negative (HR = 3.12; 95%CI 1.57–6.20, *P*-value< 0.001). While an infant’s HIV-postive result was associated with reduced maternal retention, there was no evidence of an association (HR = 1.66; 95%CI 0.56–4.91; *P*-value = 0.361).

**Table 3:** Cox regression model assessing the effect of intervention and outcome of infant HIV PCR results on the hazard of non-retention in the subgroup of mothers who received their infant’s HIV test results. Note: the mothers of 10 infants received a HIV-positive test result

Parameter	Test	Infant (***n*** = 507)		Mother (***n*** = 507)	
		Hazard ratio (95%CI)^**a**^	***P***-value	Hazard ratio (95%CI)^*^	***P***-value
**PCR results**	Positive	**3**.**12 (1**.**57,6**.**2)**	**< 0**.**001**	1.66 (0.56,4.91)	0.361
**Treatment arm**	MIP^b^ vs SOC^c^	0.88 (0.42,1.85)	0.739	1.15 (0.65,2.04)	0.625
	MIP + SMS^d^ vs SOC	0.71 (0.34,1.43)	0.338	0.84 (0.48,1.46)	0.532

^a^Adjusted for sex, delivery location, estimated gestational age (infant characteristics), parity, education level, marital status, distance to the clinic, mode of transport, and ART status prior to the study (maternal characteristics)

^b^Mother-and-infant clinics

^c^Standard of care

^d^Mother-and-infant clinics with short messaging service

Fourteen of the 693 infants (2.0%) that had HIV testing were HIV-positive (Table 4). Of the 507 mothers that received their infant’s HIV test result, 10 of their infants were HIV-positive (1.97%). There were 4, 3 and 3 HIV+ infants across MIP, MP + SMS and SOC arms, respectively. Median age of infant’s HIV-positive diagnosis was 4.0 months (IQR 3.57–4.42). Two infants (A5 and A10), who were reported as being exclusively breastfed were not retained in the study after receiving their HIV-positive diagnosis, with only one of these infants (A5) reported to have initiated ART prior to dropping out. The remaining eight infants were retained in the study for a median of 11.2 months (IQR 9.9–12.8) after receiving their HIV+ test result. Of the eight retained infants, three infants (A3, A4 and A7) were not on ART at their last follow up appointment. Notably, we were also unable to determine from the available data why these infants were not on ART.

**Table 4:** Summary of data from mothers and the 10 infants diagnosed with HIV when the mother received the infant’s HIV+ test result at the infant’s last follow-up

Infant characteristics									Maternal characteristics				
ID	Study Arm	Breastfeeding status when infant dropped out of study	Breastfeeding duration (days)	Time in study (days)	Total number of visits	Age of infant when receiving HIV+ test result (days)	Time retained in study after receiving HIV+ result (mths)	On ART at last follow-up	Parity	Marital status	Education Status	Distance from centre	ART at last follow-up
A1	1	unknown	238	496	9	230	258	Yes	2	Married	None	> 5 km	No, new dx
A2	1	unknown	387	520	8	132	420	Yes	2	Married	Completed secondary	> 5 km	Yes
A3	1	unknown	347	438	4	124	309	No	4	Married	Some primary	2 km to 5 km	Yes
A4	1	Complementary/Mixed	267	267	3	131	132	No	3	Married	Some primary	> 5 km	Yes
A5	2	Exclusive	106	106	3	104	0	Started at diagnosis	2	Divorced	Some primary	2 km to 5 km	Yes
A6	2	unknown	67	451	9	115	330	Yes	4	Married	Some primary	2 km to 5 km	No, not on ART
A7	2	unknown	116	520	10	144	374	No	4	Married	Some primary	< 2 km	No, new dx
A8	3	unknown	122	465	11	117	342	Yes	3	Married	None	> 5 km	Yes
A9	3	unknown	76	520	10	74	453	Yes	3	Married	Some secondary	2 km to 5 km	Yes
A10	3	Exclusive	73	73	2	73	0	No	3	Married	Some secondary	> 5 km	No, new dx

### Breastfeeding status of mothers that received their infant’s HIV test results

When examining the breastfeeding status of the 507 mothers in the subgroup analysis of those that received their infant’s HIV results, we found 463 infants (91.1%) were being breastfed (16% exclusively and 84% complementary/mixed) at the time of the infant’s *last recorded breastfeeding status* (Supplementary Table 3). Ten infants were also found to be HIV-positive at this time (nine of whom were exclusively breastfed and one complementary/mixed). Importantly, 493 infants were HIV negative and 449 (91.1%) of these infants were still being breastfed (65 exclusively and 384 complementary/mixed).

At *the infant’s last study follow-up,* there were no additional infant HIV infections from the infant’s last breastfeeding status (Supplementary Table 4). That is, the number of HIV-negative infants (493 infants) and HIV-infected infants (10 infants) remained consistent during this time period. Four hundred and twenty-nine infants (87%) of HIV-negative infants were still being breastfed (58 exclusive, 371 complementary/mixed) indicating that a large proportion of infants were potentially at future risk of HIV infection through continued breastfeeding after the infant’s last recorded follow-up.

## Discussion

This sub-study nested within the PRIME cluster randomized trial found mothers receiving their infant’s HIV PCR test result was associated with a 70% reduction in infant retention in the study compared with mothers not receiving their infant’s HIV test result at any given time, regardless of the study arm (*P*-value< 0.001). Receipt of an infant’s HIV-positive test result was associated with a 3.12-fold increase in infants not being retained in the study, compared with receipt of a HIV-negative result. Collectively, these findings suggest increased efforts to improve retention of infants in integrated care in the context of mothers receiving their infant’s HIV test results is needed to ensure HIV-negative infants at risk of MTCT receive follow-up HIV testing. Moreover, 87% of HIV-exposed infants were still breastfed at their final follow-up visit but were at potential risk of not being retained in the study and therefore may not have undergo repeat HIV testing 6 weeks after the cessation of breastfeeding, in line with Malawi Ministry of Heath recommendations [26]. Further, retaining infants diagnosed as HIV-positive in integrated care is needed to ensure infants receive prompt and sustained ART, thereby avoiding the high incidence of infant mortality (50%) associated with non-treatment in infants ≤24 months [15–17].

Overall coverage of infant HIV testing in rural and semirural Malawi was suboptimal, with only 71% of HIV-exposed infants receiving a HIV test < 5 months postpartum, despite Malawi guidelines recommending HIV testing of all HIV-exposed infants at 6 weeks postpartum [27]. Of the HIV-exposed infants with documented HIV test results, 74.3% of mothers received their infant’s HIV test result. In turn, this equates to 47% of all mothers receiving their infant’s HIV test result, consistent with the Diallo et al. study where ~ 50% of mothers received their infant’s HIV results in five sub-Saharan countries during 2011–2015, including Malawi [7]. Importantly, these findings suggest current estimates of incident infant HIV infections in Malawi that occur via MTCT are likely to be under-estimates of the true number.

We were unable to determine why 25.7% of mothers did not receive their infant’s HIV test result, despite mothers still being retained in the study, as this observation was after completion of the study. Possible reasons include: the testing facility did not send the result; staff failed to convey results to mothers or; staff did not document when they notified the mother. However, as the proportion of mothers receiving their infant’s HIV results were similar across all study arms, this missing data is unlikely to introduce selection bias and/or impact on our study findings. Qualitative interviewing of mothers in the PRIME study may also help to clarify why mothers did not receive their infant’s HIV results.

While 14 infants (2.0% of all infants tested) were diagnosed with HIV, only six infants were receiving paediatric ART at their last follow-up and a further infant had commenced ART at diagnosis (3.5 months postpartum) but was subsequently lost to follow-up. While we could not ascertain whether HIV-positive infants were receiving HIV treatment at another facility, other studies support the suboptimal use of ART in HIV-positive infants; in 2016, access to ART in 21 priority countries, including Sub-Saharan Africa, was ~ 51%, [6] and in 2012, Dube et al. found ART initiation in Malawi was ~ 58% [8].

Overall, retention of HIV-positive mothers and HIV-exposed infants in the study at 6 months postpartum was 95 and 86%, respectively, consistent with previous PRIME analyses using log binomial regression [10]. Notably, these figures represent a dramatic improvement from 6-month retention rates of ~ 19% for women and infants in rural Malawi in 2005 [28]. Moreover, we found infant and mother retention was 49 and 50%, respectively, at 18 months postpartum. However, we found no evidence of improved maternal or infant retention in MIP + SMS and MIP compared with SOC in contrast to a recent 3-Arm Cluster Randomized Controlled Trial in Malawi (PURE Malawi) where maternal uptake and retention in Option B+ was higher in facility- and community-based peer support models compared with SOC [29].

Similarly, mentor-mother initiatives to support and care for HIV-positive women and synchronized mother-and-baby appointments at health facilities in Kenya were associated with improved mother and infant retention, and a decrease in infant HIV infections from 13,000 in 2010 to 8000 in 2017 [30]. However, zero elimination was not achieved in Kenya, mainly due to the unreliable integration of health care/antenatal care and HIV facilities. These findings are consistent with studies in Mozambique and Uganda that found inconsistent clinical care and fragmentation across integrated clinics were driving factors for infants not being retained in integrated care [12, 13]. Whilst difficult to examine in a quantitative study, qualitative interviewing of PRIME mothers could determine whether the failure of integrated services to improve retention was the result of suboptimal delivery of integrated facilities or poor fidelity to these interventions [20].

Factors not influencing maternal or infant study retention included parity, distance to clinic, gestational birth age, delivery location or longer travel times. However, mothers who commenced ART prior to enrolment had higher infant and maternal retention than those who commenced ART during pregnancy which may suggest these mothers may have had advanced appreciation and/or understanding of the benefits of ART/adherence to healthcare services due to previous experience. Conversely, married women had a 60% increased hazard of non-retention compared with single women, suggesting a non-supportive husband or family environment may contribute to reduced maternal retention, consistent with a previous MTCT study in Malawi, [31] and a qualitative study nested within the PRIME study of HIV-infected mothers and health care providers [32].

While receiving an infant’s HIV test result was associated with reduced infant retention, consistent with other studies, [12–14] no association was found with maternal retention. Conversely, the qualitative study nested in the PRIME study found receiving an infant’s HIV negative test result was reported as being a contributing factor for maternal non-retention to HIV care, despite receiving counselling of the importance of adherence to sustained HIV care [32].

Without qualitative interview of mothers, we can only speculate why infant drop-out occurred more rapidly than maternal drop-out after mothers received their infant’s HIV test result. Of concern, mothers may be under the impression that once the infant has been tested as HIV-negative they are “safe” and no longer at risk of contracting HIV, even though the infant is still being breastfed. A further contributing factor could be extended turn-around-times between taking an infant’s dried blood spot and receiving the HIV test result as we found the median turn-around-time was 2 months, versus WHO recommendations of 4 weeks, [33] consistent with qualitative studies of HIV-infected mothers in Mozambique and Uganda where long turn-around-times were associated with increased infant drop-out in integrated services and ART inaccessibility for HIV+ infants [12, 13]. Moreover, extended turn-around times may explain why some mothers retained in the study failed to receive their infant’s HIV test result by the end of the study.

### Strengths and weaknesses of study

Strengths of this present study included the large number of participants in each of the study arms, the comprehensive data collected and the rigorous quality control checks of the entered data, especially infant HIV testing data. We also used judicious survival modelling (time-varying Cox regression) to examine the effect of exposures (the study arms and receiving infant HIV testing results) on maternal and HIV-exposed infant (or infant) study non-retention, justifying that the standard Cox model would not have been appropriate due to time-dependent nature of both of these exposures. Further, as SOC is routinely provided to HIV-infected mothers and HIV-exposed infants across different sub-Saharan countries, albeit with some variation in eligibility and ART initiation timing, [34] our study findings have the potential to be generalized to other sub-Saharan countries outside Malawi to increase HIV-exposed infant retention in integrated care.

Study limitations included the lack of data on mothers and HIV-exposed infants who were transferred or referred to other facilities for HIV care. Notably, this is a common limitation of studies examining the loss to follow-up of HIV-exposed infants as demonstrated by a 2013 meta-analysis where eight of 25 included studies from sub-Saharan Africa either did not examine the transfer of HIV-exposed infants to other HIV care services [14]. However, we believe that the HIV-exposed infants in PRIME were unlikely to be receiving HIV-related care elsewhere as not all healthcare sites in Malawi offered paediatric ART. We were also unable to determine whether infants that dropped out of the study had stopped breastfeeding and hence were no longer at risk of HIV (graduated from PMTCT program)” or infants were relocated or deceased. Consequently, these infants may have been lost to follow-up but they were not at risk of HIV infection to warrant future follow-up.

While we were unable to ascertain why 29% of HIV testing data was missing, these findings are consistent with the HIV testing rates in Malawi at the time of the study [8]. However, it is unlikely that missing HIV testing data is the result of data entry error as HIV testing data had extensive quality control checks against MoH facility HIV testing data. Nevertheless, these findings highlight that the HIV testing regimen in HIV-exposed infants in rural Malawi is suboptimal and in need of improvement.

## Conclusions

While the prevention of HIV infection through MTCT is both cost effective, [35] and saves lives, [4] infant and mother retention in the PRIME study at 18 months postpartum was 49 and 50%, respectively. Further, receiving an infant’s HIV result was associated with a 1.7-fold higher likelihood of infant non-retention, with 3.1-fold higher likelihood of non-retention when an infant’s result was HIV-positive. Rates of breastfeeding in HIV negative infants at the infant’s last follow-up were high emphasising that a high proportion of infants may be at risk of future MTCT of HIV through continued breastfeeding. Overall, these findings suggest the need for further studies to identify and address community-, family-, personal- and healthcare-related factors associated with reduced mother and HIV-exposed infant retention in integrated programs. Studies to address the impact of intensified training and increased counselling to advise breastfeeding HIV-infected mothers of the risks of future MTCT despite receipt of an infant’s HIV-negative result and the importance of retaining HIV-infected infants in HIV care are also warranted. Ultimately, improved retention in integrated HIV and healthcare services may help Malawi and other resource-limited countries in sub-Saharan Africa become a step closer to zero MTCT of HIV infection.

## Supplementary Information

**Additional file 1.**

**Additional file 2.**

**Additional file 3.**

**Additional file 4.**

## Data Availability

Restrictions apply to the availability of study data, which were used under license for the present study, and so this data is not publicly available. However, the data that support the findings of this study are available from the corresponding author on reasonable request.

## Abbreviations

ART: Antiretroviral therapy
CRT: Cluster randomized trial
HEI: HIV-exposed infant
HIV: Human immunodeficiency virus
MIP: Mother-and-infant clinic;
MIP + SMS: Mother-and-infant clinic with short messaging service
MTCT: Mother-to-child transmission;
PCR: Polymerase chain reaction
PRIME: Promoting Retention among Infants and Mothers Effectively
SOC: Standard of care
