# Supplementary material for: Receipt of infant HIV DNA PCR test results is associated with a reduction in retention of HIV-exposed infants in integrated HIV care and healthcare services: a quantitative sub-study nested within a cluster randomised trial in rural Malawi
Source: BMC Public Health. 2020 Dec 7;20:1879. doi: 10.1186/s12889-020-09973-y (PMC7720620; doi:10.1186/s12889-020-09973-y)
Supplement: Supplementary file 1 — Additional file 1. [file 12889_2020_9973_MOESM1_ESM.pdf]

Supplementary Table 1: Outline of PRIME study arms

Study arm	Services provided at one time and access point	Follow-up attempts of participants with missed visits by community health personnel	SMS reminder to community-based volunteers to follow-up participants with missed visits.
Arm 1 (MIP)	✔	✔	✗
Arm 2 (MIP+SMS)	✔	✔	✔
Arm 3 (SOC)	✗	✔	✗
