# Supplementary material for: Receipt of infant HIV DNA PCR test results is associated with a reduction in retention of HIV-exposed infants in integrated HIV care and healthcare services: a quantitative sub-study nested within a cluster randomised trial in rural Malawi
Source: BMC Public Health. 2020 Dec 7;20:1879. doi: 10.1186/s12889-020-09973-y (PMC7720620; doi:10.1186/s12889-020-09973-y)
Supplement: Supplementary file 2 — Additional file 2. [file 12889_2020_9973_MOESM2_ESM.pdf]

Supplementary Table 2: Infant HIV PCR test results of tests performed less than 2 months postpartum

No. HEIs (% of participants in study group)	MIP (n=379)	MIP+SMS (n=390)	SOC (n=298)
Tested	273 (72.0%)	299 (76.7%)	185 (62.1%)
Negative	220 (58.0%)	283 (72.6%)	165 (55.4%)
Positive	6 (1.6%)	5 (1.3%)	3 (1.0%)
Unknown	47 (12.4%)	11 (2.8%)	17 (5.7%)
