# Supplementary material for: Receipt of infant HIV DNA PCR test results is associated with a reduction in retention of HIV-exposed infants in integrated HIV care and healthcare services: a quantitative sub-study nested within a cluster randomised trial in rural Malawi
Source: BMC Public Health. 2020 Dec 7;20:1879. doi: 10.1186/s12889-020-09973-y (PMC7720620; doi:10.1186/s12889-020-09973-y)
Supplement: Supplementary file 3 — Additional file 3. [file 12889_2020_9973_MOESM3_ESM.pdf]

**Supplementary Table 3:** Breastfeeding status of the subgroup of 507 HIV-infected women that received their infant's HIV test results *at the infant's last recorded breastfeeding status.*

| **Breastfeeding status** | **HIV PCR test result** | | | **Total** |
|---|---|---|---|---|
| | **Negative** | **Positive** | **Missing** | |
| Exclusive | 65 | 9 | 0 | 74 |
| Complementary/mixed | 384 | 1 | 4 | 389 |
| *Stopped breastfeeding < 6 weeks | 12 | 0 | 0 | 12 |
| *Stopped breastfeeding > 6 weeks | 32 | 0 | 0 | 32 |
| *TOTAL* | 493 | 10 | 4 | 507 |

*Information recorded in dataset
