# Supplementary material for: Receipt of infant HIV DNA PCR test results is associated with a reduction in retention of HIV-exposed infants in integrated HIV care and healthcare services: a quantitative sub-study nested within a cluster randomised trial in rural Malawi
Source: BMC Public Health. 2020 Dec 7;20:1879. doi: 10.1186/s12889-020-09973-y (PMC7720620; doi:10.1186/s12889-020-09973-y)
Supplement: Supplementary file 4 — Additional file 4. [file 12889_2020_9973_MOESM4_ESM.pdf]

Supplementary Table 4: Breastfeeding status of subgroup of 507 HIV-infected women that received infant's HIV test results *at the date of censoring (when the infant dropped out of the study).*

**Breastfeeding status**	**HIV PCR test result**			**Total**
	**Negative**	**Positive**	**Missing**	
Exclusive	58	2	0	60
Complementary/mixed	371	1	3	375
*Stopped breastfeeding < 6 weeks	12	0	0	12
*Stopped breastfeeding > 6 weeks	32	0	0	32
Unknown	20	7	1	28
TOTAL	493	10	4	507

*Information recorded in dataset
